# Human neural stem cell-induced endothelial morphogenesis requires autocrine/paracrine and juxtacrine signaling

**DOI:** 10.1038/srep29029

**Published:** 2016-07-04

**Authors:** Chung-Hsing Chou, Michel Modo

**Affiliations:** 1 University of Pittsburgh, Department of Radiology, Department of Bioengineering, McGowan Institute for Regenerative Medicine, Pittsburgh, USA; 2 Kings College London, Department of Neuroscience, London, UK; 3 Tri-service General Hospital, Department of Neurology, National Defense Medical Centre, Taipei, Taiwan

## Abstract

Transplanted neural stem cells (NSC) interact with the host brain microenvironment. A neovascularization is commonly observed in the vicinity of the cell deposit, which is correlated with behavioral improvements. To elucidate the signaling mechanisms between human NSCs and endothelial cells (ECs), these were cocultured in an *in vitro* model in which NSC-induced endothelial morphogenesis produced a neurovascular environment. Soluble (autocrine/paracrine) and contact–mediated (juxtacrine) signaling molecules were evaluated for two conditionally immortalized fetal NSC lines derived from the cortical anlage (CTXOE03) and ganglionic eminence (STROC05), as well as an adult EC line (D3) derived from the cerebral microvasculature of a hippocampal biopsy. STROC05 were 4 times as efficient to induce endothelial morphogenesis compared to CTXOE03. The cascade of reciprocal interactions between NSCs and ECs in this process was determined by quantifying soluble factors, receptor mapping, and immunocytochemistry for extracellular matrix molecules. The mechanistic significance of these was further evaluated by pharmacological blockade. The sequential cell-specific regulation of autocrine/paracrine and juxtacrine signaling accounted for the differential efficiency of NSCs to induce endothelial morphogenesis. These *in vitro* studies shed new light on the reciprocal interactions between NSCs and ECs, which are pivotal for our mechanistic understanding of the efficacy of NSC transplantation.

Human neural stem cell (NSC) transplantation is emerging as a potential therapeutic strategy for stroke^1^. A major advantage of cell lines, such as ReN001 (CTXOE03), is that each patient with chronic stroke in a phase II clinical trial (NCT02117635) receives a homogenous well-characterized population of cells that can be produced on an industrial scale^2^. In a preclinical efficacy study using CTXOE03, behavioral improvements were correlated with astrocytic differentiation of transplanted cells, as well as neovascularization at the site of injection^3^. Indeed, CTXOE03 has a robust angiogenic phenotype^4^,^5^, but other NSC lines, such as STROC05, also exhibit neovascularization at the site of injection^6^. Others also reported an interdependent increase in angiogenesis and neurogenesis after a stroke^7^,^8^,^9^, with the formation of a vascular network being associated with better NSC survival^10^. There is also an indication that systemic blocking of neovascularization is preventing behavioral recovery after NSC transplantation^11^, potentially suggesting that endothelial cells (ECs) are the main, even though indirect, therapeutic effector^12^.

Nevertheless, an association between neovascularization and behavioral recovery does not imply causality. Indeed, most biological systems are the product of a complex interplay between different types of cells influencing each other^13^, hence a more complicated mechanistic interaction with synergistic properties might emerge. Elucidating the neurobiological mechanisms underlying NSCs’ therapeutic efficacy therefore needs to consider continuing autocrine, paracrine and juxtacrine interactions between NSCs and ECs, including the formation of novel blood vessels (i.e. vasculogenesis). Identifying individual signals that can be manipulated to modulate efficacy is therefore indispensable to disentangle the causal cascade. Pivotal factors in blood vessel formation have been identified in vasculogenesis in the developing brain, the neural stem cell niche, as well as tumor-induced angiogenesis^14^,^15^. Autocrine and paracrine factors, such as vascular endothelial growth factor (VEGF), brain derived neural growth factor (BDNF), basic fibroblast growth factor (bFGF), stromal derived factor-1α (SDF-1α), platelet derived growth factor (PDGF), angiopoietin (Ang) and transforming growth factor-β1 (TGF-β1), influence the vascular environment by diffusion, hence affecting multiple cells in the vicinity of their release. In contrast, juxtacrine factors, such as vitronectin, fibronectin, laminin, collagen I & IV, hyaluronic acid (HA), aggrecan, neurocan, thrombospondin, nidogen and brain link protein 1 (Bral1), affect neurovascular interactions by close contact cell-to-cell or extracellular matrix (ECM)-to-cell signaling. Indeed, a synergistic effect between autocrine/paracrine and juxtacrine factors is required to produce endothelial morphogenesis and enhance neuronal differentiation of NSCs^16^.

To gain a mechanistic understanding of interactions between NSCs and brain ECs, autocrine/paracrine (i.e. soluble factors), as well as juxtacrine targets, were investigated in an *in vitro* coculture model of the neurovascular environment using human cerebral microvascular ECs (D3) and two clinical-grade human NSC lines (STROC05 & CTXOE03)^16^. NSCs facilitated endothelial morphogenesis (EM) in a reciprocal relationship with neuronal differentiation and allowed us to measure autocrine/paracrine and juxtacrine signals, as well as the corresponding receptors. A concentration-dependent effect was evident for both cell lines, but STROC05 were significantly more efficient to induce EM, hence forming better-defined vessel-like structures (VLS). Autocrine/paracrine and juxtacrine signaling provides essential factors through EM, but neither provides a sufficient factor to induce VLS, indicating that NSCs’ efficiency cannot be reduced to a single factor. Nevertheless, inhibition of a single factor is sufficient to prevent VLS formation by interfering with EM.

## Results

### NSCs differentially induce VLS in a concentration-dependent fashion

**Figure 1:**
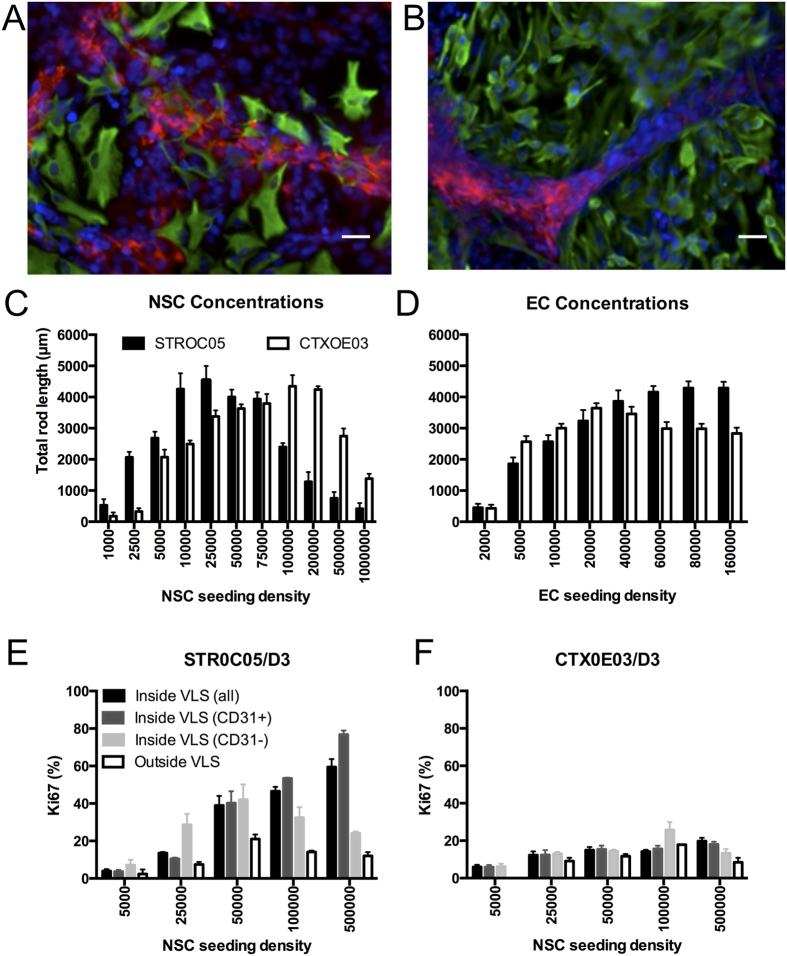
Endothelial morphogenesis is dose-dependent. Endothelial morphogenesis is the process that occurs in ECs (red = CD31) when cocultured with striatal STROC05 **(A)** or cortical CTXOE03 **(B)** NSCs (green = nestin), resulting in vessel-like structures (VLS, blue = DAPI). Scale bar = 50 μm. **(C)** Endothelial morphogenesis, as measured by total rod length of vessel-like structures^16^, exhibits a dose-dependent Gaussian function in relationship with NSCs, with fewer STROC05 cells (1:16; NSC:EC ratio) being required to induce VLS compared to CTXOE03 (1:4). **(D)** In contrast, dose-dependence of ECs follows a linear curve up to a ratio of 1:1 (40,000 cells each) for both STROC05 and CTXOE03 before leveling off. STROC05 were significantly more efficient in forming VLS with higher ratios of EC to NSC. **(E**,**F)** The proliferation rate of ECs was predominantly affected by STROC05 cells in a linear fashion without evidence of a leveling off **(E)**, whereas CTXOE03 had little influence on proliferation **(F)**. Conversely, ECs only influenced the STROC05 proliferation at a seeding density of 50,000 cells. Data represent the mean and error bars indicate 1 standard deviation.

NSCs induced endothelial morphogenesis (EM) of endothelial cells (ECs), resulting in the formation of VLS *in vitro*. VLS are characterized by ECs forming a tubule-like shape surrounding a NSC-based “neuropil patch”. This plexus of VLS is induced by both STROC05 (Fig. 1A) and CTXOE03 (Fig. 1B) in an inverted U-shape concentration-dependent fashion (Fig. 1C). Although both NSC lines can produce a similar level of VLS, STROC05 were 4x more efficient in this process than CTXOE03. Increasing the number of ECs in coculture led to a linear increase in VLS formation before leveling off for STROC05 and leading to a small, yet significant regression, for CTXOE03 (Fig. 1D). STROC05 produced a very significant concentration-dependent increase in EC proliferation (Fig. 1E), whereas there was little change in proliferation in CTXOE03-D3 cocultures (Fig. 1F). STROC05 and CTXOE03, both NSC lines, hence differ significantly in their effect on EM, as well as EC proliferation.

**Figure 2:**
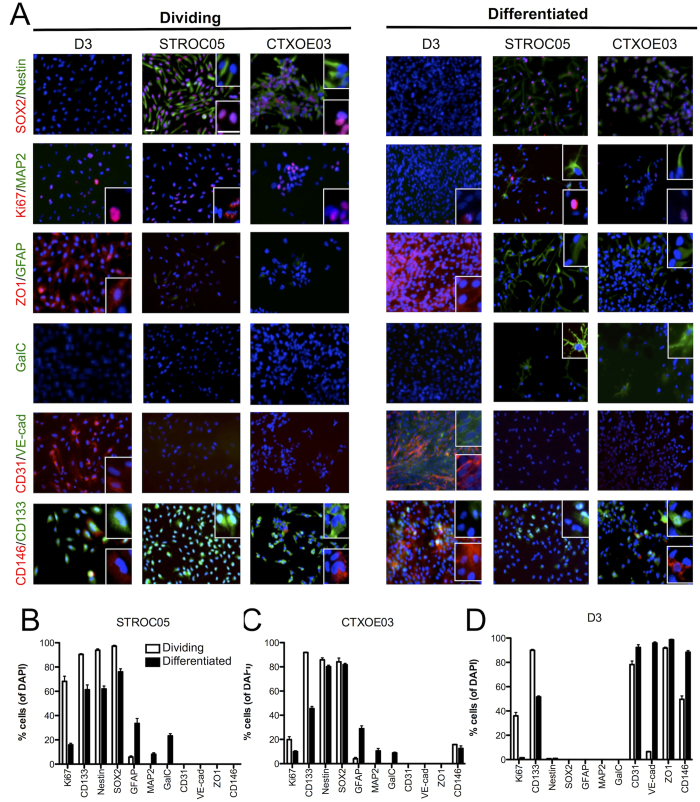
Phenotypic characterization of undifferentiated and differentiated cell lines. **(A)** Immunocytochemistry of dividing, undifferentiated and differentiated ECs (D3) and NSCs (STROC05 & CTXOE03). Scale bars represent 50 μm. Expression of phenotypic markers in STROC05 **(B)**, CTXOE03 **(C)** and D3 cells **(D)** in monoculture. Data represent the mean and error bars indicate 1 standard deviation.

**Figure 3:**
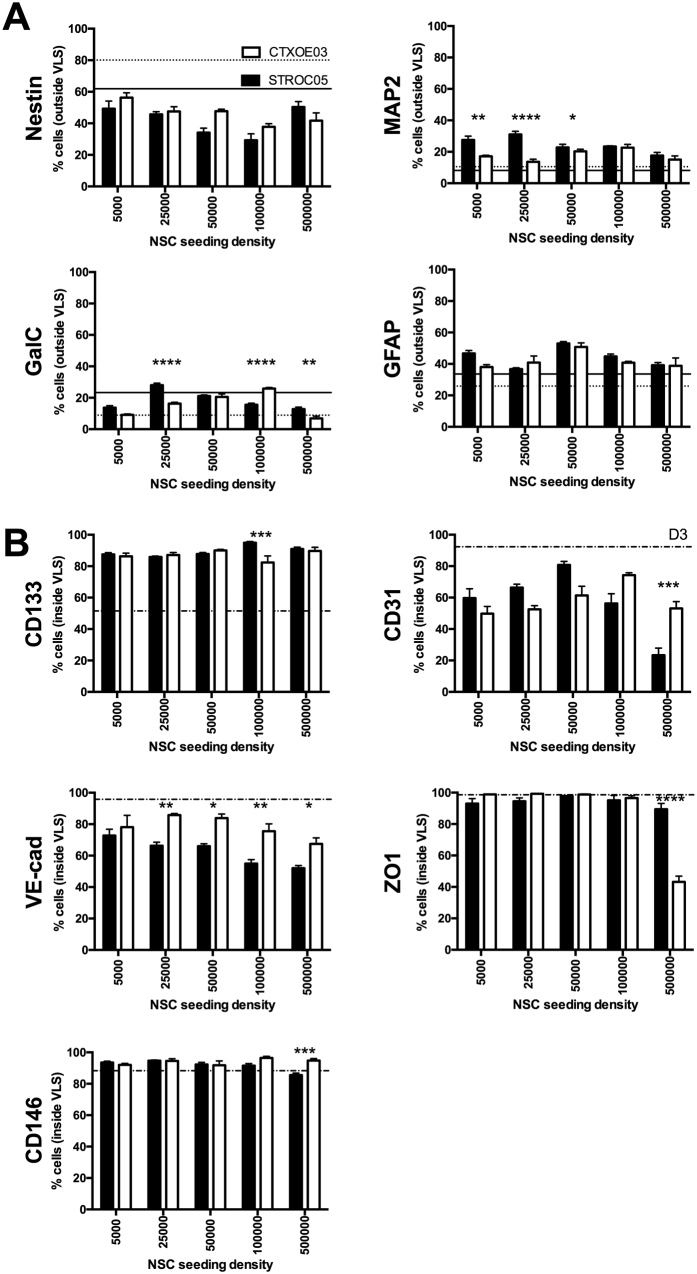
Phenotypic differentiation of NSCs and ECs is affected by endothelial morphogenesis. **(A)** NSCs cocultured with ECs significantly increased differentiation in relation to monoculture (solid and dashed lines for STROC05 and CTXOE03 respectively). Especially STROC05 increased neuronal differentiation (MAP2+ cells). Seeding numbers of NSC at which endothelial morphogenesis was highest (25,000 for STROC05 and 100,000 for CTXOE03) exhibited the greatest effect on NSC differentiation indicating a potential coupling of the two processes. **(B)** Conversely, in NSC/EC coculture, mature EC markers CD31 and VE-Cadherin were reduced in D3 cells compared to EC monocultures. A dose-dependent effect for STROC05 and CTXOE03 was evident for both markers. ZO1 and CD146 were comparable with only a difference being evident at the highest coculture ratio. Consistent with the decrease in mature ECs markers, CD133 expressed in immature ECs was almost twice as high. Data represent the mean and error bars indicate 1 standard deviation. (*)p < 0.05, (**)p < 0.01, (***)p < 0.001, (****)p < 0.0001.

To establish if NSCs and ECs’ interactions affect phenotypic differentiation, immunocytochemical markers for dividing undifferentiated and non-dividing differentiated cells were first quantified in standard monocultures to provide a baseline characterization (Fig. 2A). As expected, a general shift from proliferative (Ki67) and undifferentiated markers (CD133, Nestin, SOX2) to more mature phenotypic markers of NSCs (GFAP, MAP2, GalC) and ECs (CD31, VE-Cadherin, ZO1, CD146) was observed for STROC05 (Fig. 2B), CTXOE03 (Fig. 2C) and D3 cells (Fig. 2D). These baseline measures served as comparison for cocultures to determine effects on neural differentiation markers in NSCs (Figure S1A) and endothelial markers in ECs (Figure S1B). Coculture of NSCs with ECs significantly reduced the presence of Nestin-expressing undifferentiated NSCs in a concentration-dependent, but cell line independent, fashion (Fig. 3A, Table S1). A concentration-dependent effect was also evident for neuronal (MAP2+) differentiation. Indeed, neuronal differentiation in coculture was enhanced 3-fold for STROC05 and 2-fold for CTXOE03 at the same concentration that resulted in optimal VLS formation (seeding densities of 25,000 STROC05; or 100,000 CTXOE03 NSCs per well). Maximum neuronal differentiation therefore coincides with optimal EM. However, all EC concentrations had a significant effect on neuronal differentiation compared to monoculture.

In contrast, oligodendrocytes (GalC+) were equivalent to monocultures only at an optimal concentration with lower differentiation levels at sub-/supra-optimal concentrations for STROC05. CTXOE03, in contrast, revealed an up to 2-fold increase in oligodendrocytic and astrocytic differentiation. Reciprocal signaling is also evident in EC phenotypes (Figure S1B). The presence of NSCs actually preserves an undifferentiated phenotype in ECs with 35% more cells expressing CD133 for all concentrations (Fig. 3B), which is consistent with an increase in EC proliferation. The preservation of an undifferentiated phenotype results in a decrease in CD31 and VE-Cadherin, both markers of mature ECs. CD31 exhibited an inverted U-shape curve that revealed a concentration and NSC line interaction akin to that observed on EM and neuronal differentiation. Although a concentration and cell line effect was also evident for VE-Cadherin, this effect was linear with higher concentrations of NSCs resulting in an up to 45% reduction of VE-Cadherin+ cells. Surprisingly, ZO1, a marker of cell-to-cell junctions found at the blood-brain barrier was only significantly reduced at the highest concentration of CTXOE03 cells. These results suggest that NSCs exert a concentration-dependent and reciprocal effect on ECs. However, there are also signaling differences between STROC05 and CTXOE03 that are not due to concentration. These effects are likely to be mediated through differences in secreted (autocrine/paracrine) and/or cell-to-cell (juxtacrine) signaling factors.

### Sequential regulation of autocrine/paracrine signaling

**Figure 4:**
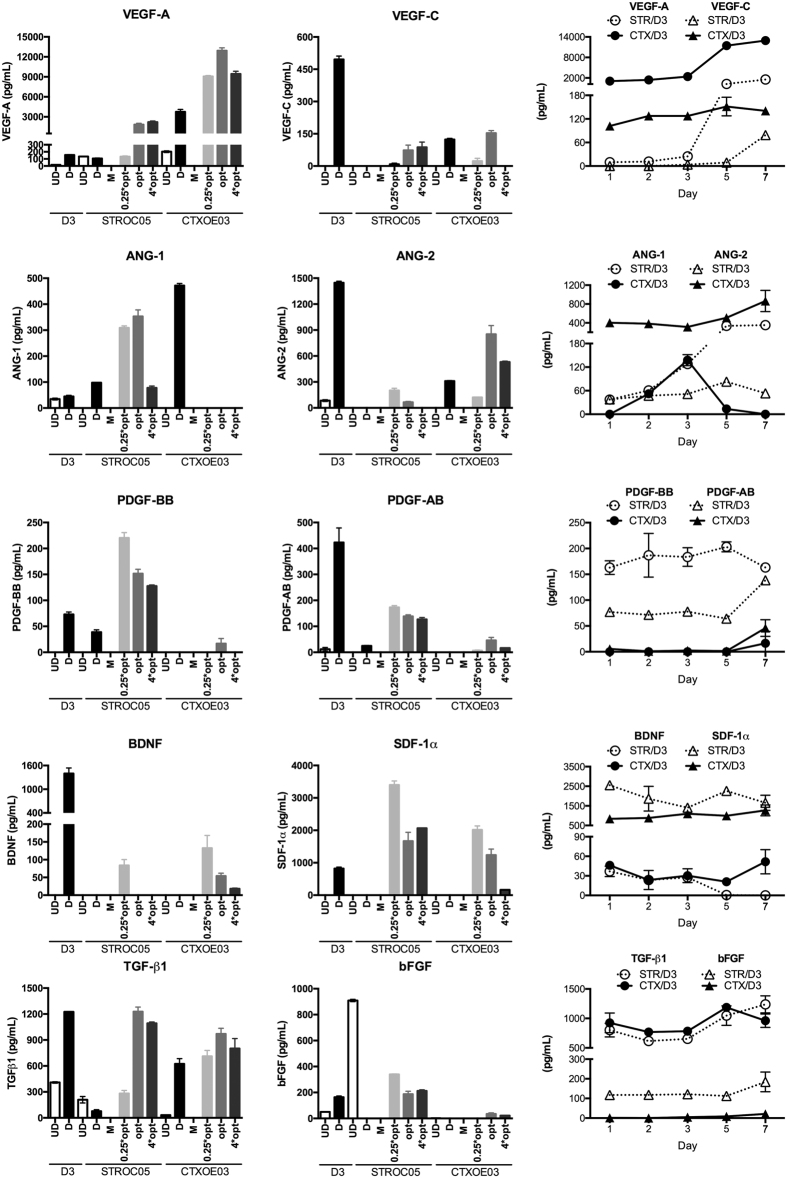
The secretome of NSCs and ECs undergoing endothelial morphogenesis. Secreted factors from undifferentiated (UD) and differentiated (D) D3, STROC05 and CTXOE03, as well as plain media (M), were measured using ELISAs. Additionally, 7 day co-cultures at a ¼x (0.25*opt), optimal (opt), or 4x optimal (4*opt) ratio, as well as the evolution of factor secretion of the optimal ratio over time were assessed. All factors tested here were secreted from differentiated ECs, but were mostly absent in dividing undifferentiated D3. In contrast, monocultures of NSCs under proliferative or differentiated conditions mostly released low levels of the factors, predominantly factors associated with a stabilization of the vasculature (e.g. TGF-β1, ANG-1). CTXOE03 however expressed high levels of VEGF-A, especially when differentiated. A dramatic change in the level and profile of factor release is observed after coculture. Especially BDNF, VEGF-A, PDGF, ANG-1, TGF-β1 and bFGF showed distinctive cell line profiles in coculture, indicating that NSCs potentially exert differential effects on EC behavior through the interaction of secreted factors. The temporal profile further indicates how these secreted factors evolve from in concurrence with endothelial morphogenesis and cellular differentiation. Data represent the mean and error bars indicate 1 standard deviation.

Secreted factors that act in an autocrine and/or paracrine fashion are released from cells and can be assayed in the supernatant. In a coculture, cell signaling and its ensuing behavior are therefore dependent on the phenotypic state of cells as they initiate interactions. Pivotal secreted factors were measured using ELISAs of supernatant from monocultures of dividing undifferentiated and non-dividing differentiated cells and compared with cocultures that were either at an optimal concentration or sub-/supra-optimal conditions (Fig. 4, Table S2). At the top of the canonical cascade of NSC:EC interactions is VEGF-A. Undifferentiated ECs secreted minute amounts of VEGF-A that are increased upon differentiation. In contrast, NSCs, and especially CTXOE03 express higher levels of VEGF-A. This is dramatically increased upon CTXOE03 differentiation, but even more so in coculture with differentiated ECs. In coculture, STROC05 upregulated VEGF-A release ~16,000 times. Nevertheless, in absolute terms CTXOE03 released the highest amount of VEGF-A with ~12,000 pg/mL. In both cocultures, this dramatic increase in VEGF-A release is seen on day 5 with little change compared to baseline beforehand. Although VEGF-C was significantly upregulated in differentiated ECs, coculturing lead to a significant and stable decrease in release. However, VEGF-C was more prominently present in CTXOE03 co-cultures with no detectable levels in STROC05 co-cultures until day 7. VEGF-D was not detectable in any monocultures and cocultures.

Ang-1 release, a critical factor in vessel maturation, was significant in differentiated NSCs, but not undifferentiated NSCs, although it was equally present in dividing and differentiated ECs. Coculture lead to a gradual increase in the presence of STROC05, but only a transient increase in CTXOE03 cells at day 3. Ang-2 is known to disrupt vascularization and was stably present over time in co-culture, but was 8x more abundant in the presence of CTXOE03 than STROC05. This is in contrast with PDGF-AB and -BB (modulating cell proliferation and promoting vessel maturation) that are consistently higher in STROC05 cocultures and only detectable in CTXOE03 cocultures at day 7. BDNF (a survival factor) and SDF-1α (controlling cell migration) are only present in differentiated ECs and are consistently stable in coculture with the exception of a BDNF decrease in STROC05 coculture from day 5. The final step in the formation of a stable vessel structure is dependent on the synergistic effects of TGF-β1 and bFGF. Although TGF-β1 was equally present in both coculture conditions, only STROC05 coculture released bFGF. Timing of bFGF, however, controls its function with early release of bFGF in the angiogenic cascade promoting cell proliferation, which is also observed in ECs in the STROC05 coculture. The controlled co-release of TGF-β1 is hence likely serving as a switch to vessel stabilization rather than cell proliferation.

### Differential localization of juxtacrine signals

To exert a precise spatial and temporal signaling control, juxtacrine factors require close contact to control tissue cytoarchitecture, as well as the formation and maintenance of tissue compartments. Using immunocytochemistry, neuropil- and vasculature-associated molecules can be contrasted for their role in NSC-induced endothelial morphogenesis. The basement membrane defines the signaling interface between the neuropil and vasculature. VLS exhibited the formation of a basement membrane that was characterized by its typical ECM molecules, notably vitronectin, fibronectin, collagen I, collagen IV, as well as laminin (Figure S2). Within the “neuropil”, cells only expressed laminin. In contrast to vitronectin and laminin, neither collagen I & IV, nor fibronectin was present in undifferentiated or differentiated NSCs. Undifferentiated D3 ECs strongly expressed fibronectin and laminin, with a small number of cells also expressing collagen I & IV and vitronectin. Upon D3 differentiation, all these molecules were strongly expressed, indicating their tight association with the maturation of a vasculature.

Neuropil-associated molecules were mostly present within the neuropil, but also associated with VLS (Figure S3). Indeed, hyaluronic acid (HA) was evident on NSCs in the “neuropil”, but was also strongly present in VLS. Undifferentiated and differentiated D3 more strongly expressed HA compared to both NSC lines. Aggrecan was only weakly present in neuropil, but CTXOE03 expressed it under both undifferentiated and differentiated conditions, with significantly lesser expression in D3 and none in STROC05. A similar expression pattern was also evident for neurocan, with some clear cellular expression in CTXOE03 in coculture. Thrombospondin, a molecule often associated with reactive astrocytes, was strongly present in cocultures. Although it colocalized with NSCs, this was only in NSCs that were in proximity to ECs. Indeed, ECs also expressed thrombospondin in coculture, as well as in their undifferentiated and differentiated state. Interestingly, in ECs thrombospondin was morphologically distinct compared to NSCs, with a fiber-like morphology. In contrast to all other molecules, nidogen did not exhibit a differential expression in any of the conditions examined with a strong cytoplasmic appearance and some extracellular punctate presence. Finally, Bral1 was weakly expressed in STROC05 and downregulated in differentiated CTXOE03 cells. Interestingly, it was upregulated in differentiated D3 monoculture, but mostly absent after co-culturing with either NSC lines.

### Autocrine/paracrine and juxtacrine receptors are present inside and outside VLS

**Figure 5:**
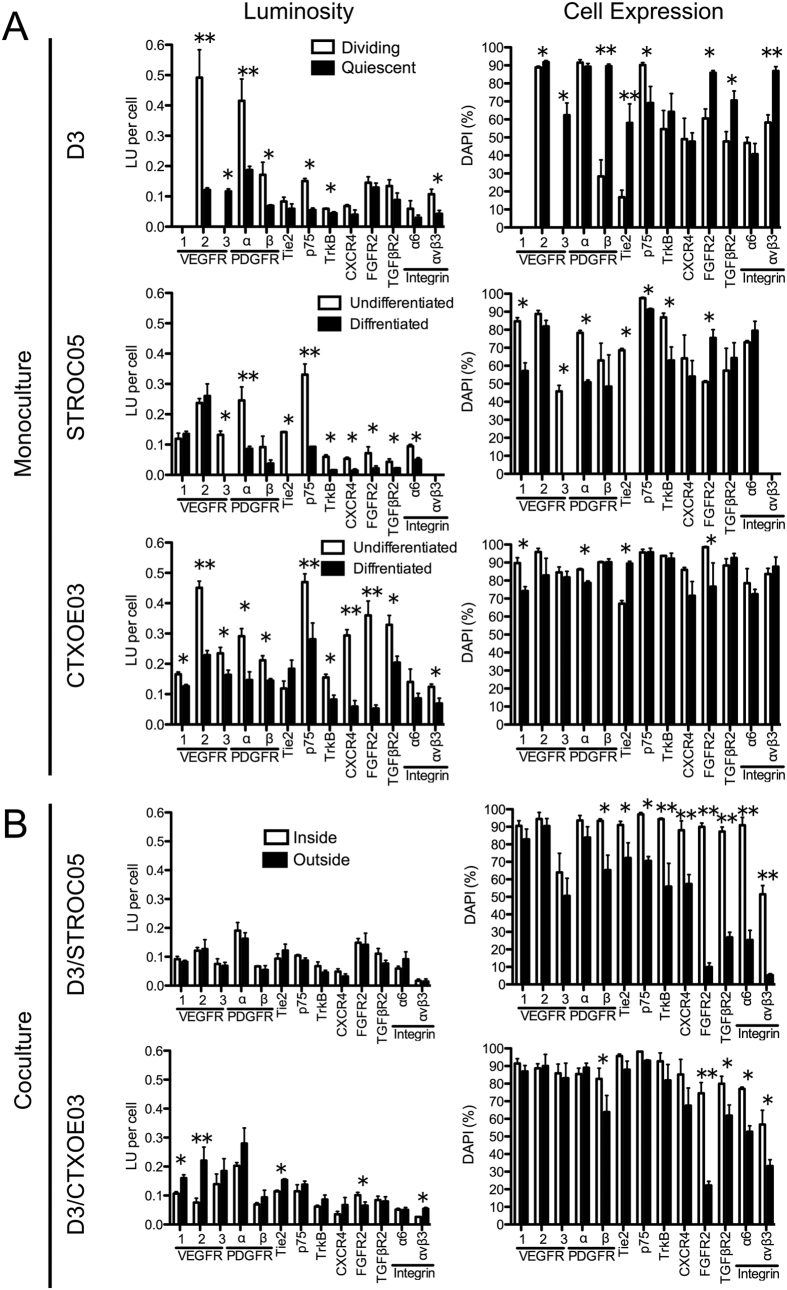
Quantification of receptor expression. Receptor expression was quantified by measuring total amount of fluorescence in the channel reflecting the antibody used to detect a given receptor divided by the number of cells present within the same field-of-view (luminosity). Additionally, the number of cells expressing a particular receptor in relation to the total number of cells per field-of-view (cell expression) were also counted. Receptor expression was quantified for cells grown in monoculture (undifferentiated and differentiated conditions) **(A)**, as well as for those grown in coculture (inside and outside VLS) **(B)**. Data represent the mean and error bars indicate 1 standard deviation. (*)p < 0.05, (**)p < 0.01.

Signaling factors are, nevertheless, dependent on binding to an appropriate receptor on their target cell (Figure S4). In monoculture, key differences in receptors were evident (Fig. 5A). Specifically within the VEGF signaling family, VEGFR1 was not present on ECs, but was expressed highly in 80–90% of undifferentiated STROC05 and CTXOE03 cells with a slight reduction upon differentiation. Although VEGFR3 was present in ~80% of undifferentiated and differentiated CTXOE03, only ~45% of undifferentiated STROC05 and 60% of differentiated D3 expressed this receptor. In contrast, VEGFR2, the receptor for VEGF-A was presented in ~90% of all cells examined potentially highlighting its pivotal role in angiogenesis. PDGF receptors were present in all cell types and conditions, although there was some variation in the number of cells expressing the α or β form of the receptor. Tie2, the receptor for ANG1, was significantly downregulated upon STROC05 differentiation, but upregulated in both CTXOE03 and D3 cells (p < 0.01). Interestingly, αvβ3 was not present in monocultures of STROC05.

Upon coculture, a key distinction in receptor mapping was between cells inside VLS and those outside (i.e. the putative neuropil) (Fig. 5B). All 3 VEGFR were present in most (~90%) cells inside and outside of VLS, with the exception of VEGFR3 in STROC05 co-cultures only being present in 50–60% of cells (p < 0.05). PDGF receptors were equally present in both cocultures and FGFR2 was significantly (p < 0.01) downregulated outside VLS. However, in contrast to CTXOE03 cells, Tie2, p75, TrkB, CXCR4, TGFβR2, α6 and αvβ3 were present in significantly fewer cells outside the VLS in STROC05 co-cultures (p < 0.01). Having mapped putative signals and their receptors hence afforded to probe mechanistically if these factors are necessary for VLS formation by interfering with their signaling.

### Preventing the generation of VLS

**Figure 6:**
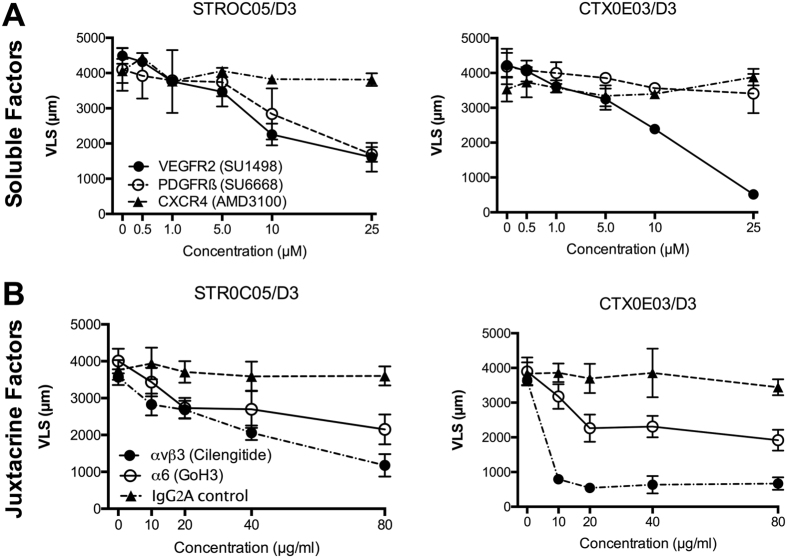
Pharmacological blocking of endothelial morphogenesis. **(A)** Blocking of secreted factors revealed that VEGFR2 signaling is essential for endothelial morphogenesis in both NSC lines. In contrast, a PDGFRβ inhibitor was only causally involved for STROC05. A CXCR4 antagonist exerted no effect. **(B)** Cilengitide, a RGD pentapeptide inhibitor against receptor integrin αvβ3, prevented endothelial morphogenesis in both cell lines, but was more potent in the D3/CTXOE03 condition. Blocking of α6 was less effective than αvβ3, but was involved in molecular interactions with ECs for both NSC lines. An IgG2A control antibody did not exert any influence on endothelial morphogenesis. Data represent the mean and error bars indicate 1 standard deviation.

The roles of paracrine signaling factors in generating neurovascular networks were assayed by blocking receptors. Specifically, VEGFR2 inhibitor SU1498, PDGFRβ inhibitor SU6668, and CXCR4 antagonist AMD3100 were added separately into cocultures (Figure S5A). The VEGFR2 inhibitor significantly inhibited VLS formation in both D3/STROC05 and D3/CTXOE03 cocultures (Fig. 6A, Table S3). Nevertheless, a significant reduction of VLS formation caused by the PDGFRβ inhibitor could only be observed in D3/STROC05, rather than D3/CTXOE03 coculture. A CXCR4 antagonist blocking the potential migration of cells did not interfere with VLS induction by either NSC line.

Interferences with juxtacrine factors was achieved using Cilengitide, a RGD pentapeptide inhibitor of integrin αvβ3, GoH3-blocking antibody against integrin α6, and IgG_2_A isotype antibody as a negative control (Figure S5B). The αvβ3 inhibitor significantly inhibited VLS formation in both D3/STROC05 and D3/CTXOE03 cocultures (Fig. 6B, Table S3), but a more pronounced effect (at a lower dose) was evident for CTXOE03 cells. A significant and equivalent reduction in VLS formation caused by the α6 inhibitor could also be seen in both D3/STROC05 and D3/CTXOE03 cocultures. In contrast, the IgG_2_A control antibody did not significantly affect the formation of VLS. The results hence demonstrate that key autocrine/paracrine signaling factors and their receptors can be identified in an *in vitro* model of neuro-vascular interactions and that these can be probed in a mechanistic fashion to determine their involvement in the formation of new vessel-like structures.

## Discussion

**Figure 7:**
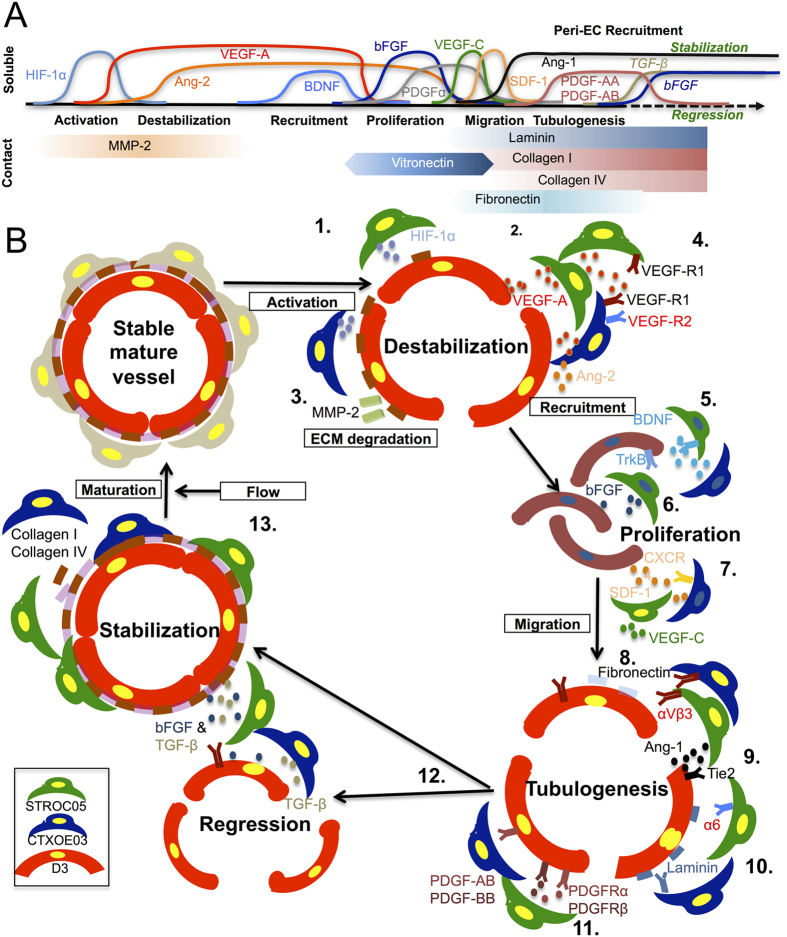
Schematic overview of vascular morphogenesis and the role of NSCs. **(A)** Sequential regulation of soluble factors and ECM molecules mediates the development of vascular morphogenesis and maintains neurovascular networks. The canonical cascade of factors indicates that each factor plays a key role in guiding sequential processes. There are fewer and more potent initiating factors compared to a greater interplay between factors in establishing a mature vessel. **(B)** CTXOE03 and STROC05 maintain or induce new vessel growth through autocrine/paracrine and juxtacrine interactions. 1.) NSCs are induced to release VEGF-A by HIF-1α^36^,^47^ as a result of hypoxia. 2.) VEGF-A destabilizes the vasculature. CTXOE03 released VEGF-A, but is also responding to it in an autocrine fashion as it expresses VEGFR2, whereas STROC05 produces VEGF-A expressing less the corresponding auto-receptor. 3.) Degradation of the basement membrane by MMP-2 signaling ensues. 4.) Recruitment of ECs from the vessel is induced by both NSC lines. 5.) BDNF is released from both NSCs lines and ECs express TrkB receptors, STROC05 and CTXOE03 also expresses the auto-receptor. 6.) NSCs, as well as ECs, secreting bFGF induce proliferation. 7.) Migration of cells is promoted by SDF-1 secreted from NSCs with ECs expressing the corresponding CXCR4 receptor. VEGF-C is only released from STROC05. 8.) Tubulogenesis is influenced by the expression of the ECM molecules vitronectin and fibronectin, providing juxtacrine motives for NSCs to more tightly control ECs’ position, as opposed to more diffusely influential paracrine signaling. 9.) Ang-1 secreted from NSCs further influences the stabilization of the tubular organization. 10.) This process is more heavily influenced by ECM molecules, such as laminin and its α6 receptor. 11.) PDGF signaling can causally influence tubulogenesis (as in the case of STROC05), but alternative signaling can obviate its causal role (see CTXOE03). 12.) Once a tubule is formed, the joint signaling of bFGF and TGF-β1 can determine if a stable vessel is being formed or if the structure is simply regressing. 13.) Further deposition of collagens allows maturation of the vessel, but only the presence of flow will eventually inflate a lumen and warrant the persistence of the structure.

An angiogenic response of the vasculature (Fig. 7A) is emerging as an important factor in the therapeutic efficacy of transplanted NSCs. Unlike pharmacological agents, NSCs and ECs interact in a responsive fashion to each other in a signaling cascade, involving both soluble secreted factors that act in an autocrine and paracrine fashion, but also contact-mediated juxtacrine factors that result in a very locally-controlled signaling. This cascade defines different crucial processes required to promote the creation of a new neuro-vascular environment. We here demonstrate that this signaling cascade that is induced by NSCs organizes ECs into VLS that define a “neuropil” separated by a basement membrane from the “vascular plexus”, but also produces a dramatic shift in the neural differentiation of NSCs. This *in vitro* assay afforded the temporal evaluation of autocrine/paracrine and juxtacrine factors involved in this cascade and allowed us to investigate the neuro-vascular signaling cascade underlying the interaction between NSCs and ECs.

### A cascade of neuro-vascular signaling

ECs by themselves do not form VLS, but are dependent on induction of this process by NSCs^16^. This induction is dependent on the NSC:EC ratio following an inverted U-shape function, revealing the importance of dose to the observed effects. However, the propensity of VLS induction exhibited a dramatic difference between STROC05 and CTXOE03, with STROC05 requiring 4 times less cells. At their optimal concentrations, an up to 3 fold increase in neuronal differentiation of NSCs could also be observed, further highlighting the interdependence that these two types of cells exert on each other. Indeed, there is accumulating evidence that factors, such as VEGF, PDGF and EGF, traditionally linked to vascular development, are also pivotal in neural development^17^. The potent effects ECs exerted on NSCs also caution about the predictive value and interpretation of monoculture experiments to determine putative therapeutic effects of NSCs, as their molecular profile and behavior is markedly different^18^. Uncovering key differences in their autocrine/paracrine and juxtacrine signaling in response to the presence of ECs hence provides a means to determine potential mechanisms by which these cells produce a neovascularization upon implantation into the peri-infarct stroke area, but also their potential to organize a novel neurovascular environment.

The organization of a neurovascular environment results from endothelial morphogenesis that is induced by NSCs, and it can be regarded as the product of a cascade of signaling interactions with ECs. In this process, both NSCs and ECs secreted factors that control their own (autocrine) function, as well as other surrounding cells (paracrine). The most potent factor here was VEGF-A, which was dramatically upregulated in both NSC lines in presence of ECs. There is increasing evidence that secretion of VEGF-A is involved in behavioral efficacy of NSCs transplanted in stroke^11^ and that VEGF-A by itself can promote behavioral improvements^19^, but this is unlikely to be due to neovascularization per se, rather than an indirect effect on NSCs or host neurons. CTXOE03’s release of VEGF-A was dramatically higher compared to STROC05, potentially driving its high angiogenic potential^4^,^5^. The high doses of VEGF generated here in coculture suggest intussusception, rather than sprouting angiogenesis, as a potential mechanisms underlying the observed *in vivo* neovascularization^20^. The greater efficiency of STROC05 to induce VLS indicates that neovascularization is not merely the product of destabilizing blood vessels and promoting ECs’ proliferative response, which are the key processes induced by VEGF-A, but also newly formed vessels, which require organization into tubules and stabilization^21^. CTXOE03 inefficiently secreted Ang-1, PDGF-AA and -AB in comparison to STROC05, indicating that it is less efficient in these two processes. Crucially, the secretion of Ang-1 and PDGF-AB increased as VLS were forming in D3/STROC05 cocultures indicating the importance of the evolution of signaling between ECs and NSCs to control particular processes in vasculogenesis. Hence CTXOE03 is a very potent inducer of an angiogenic response, but STROC05 is more efficient in forming stable VLS. For NSCs to induce a stable neurovascular environment, a shift from induction to maintenance signaling is required to ensure that no vessel regression occurs^22^.

Although secreted factors exert a key influence on the local environment, it is juxtacrine factors that provide the fine-tuning that leads to EM by organizing close cell-to-cell interactions^21^,^23^, as well as the formation of the BM that defines the intersection between the neuropil and vascular environment^24^. VLS developed a BM that involved early markers, such as vitronectin and fibronectin, but also more mature ECM markers, such as collagen I and IV, as well as laminin^25^. Although a BM formed in the presence of both NSC cell types, fibronectin and vitronectin, the earliest molecules in the formation of BM, were more evident in CTXOE03 co-cultures, whereas collagen I and IV, which are more mature markers were defining VLS in STROC05 coculture, potentially again indicating that more stable VLS are being formed. The BM also influences the interaction with the neuropil, especially astrocytes which extend end feet onto the VLS and here also performed a pericyte function^26^. Indeed, almost 20% of CTXOE03 expressed CD146, a marker typically found in pericytes^27^. Others have also reported the expression of tight junction molecules in undifferentiated NSCs^28^. Especially neurosphere preparations appear to potentially promote the adaption of endothelial cell characteristics of NSCs^29^, including the contribution^30^ or formation of VLS^31^. However, no VLS formation or expression of tight junction molecules was evident here in both NSC lines. The plasticity of NSCs to form VLS and express endothelial-associated markers might hence be a reflection of NSCs that are less developmentally mature than those used here, but could also be a reflection of specific culture conditions. Ideally, incorporation of a fluorescent marker will help to track NSCs and determine their phenotypic behavior. As the cells used here are of clinical grade, we nevertheless refrained from this approach to preserve their characteristic phenotype.

In contrast to VLS, neuropil patches mostly revealed aggrecan, neurocan, thrombospondin, hyaluronic acid, nidogen and Bral1. The deposition of these ECM molecules was increased in cocultures compared to monocultures, further indicating that ECs also contribute to the maturing process of the neuropil (e.g. formation of peri-neuronal nets and axon initial segments) and provide important instructive signals to shape the neural compartment of the neurovascular unit^32^. Although all these signaling elements influence communications between NSCs and ECs, understanding their mechanism requires a distinction between necessary conditions, i.e. absence of the factor prevents the formation of VLS, versus sufficient conditions in which the factor influences the overall efficiency of the process, but does not prevent VLS formation.

### Necessary versus sufficient vasculogenic signaling

As VLS formation is an orchestrated process, interferences with key events by blocking the pivotal signal will disrupt the signal cascade and reveal necessary factors in endothelial morphogenesis. Blocking of VEGFR2 (VEGF-A and -C receptor), as well as αvβ3 (vitronectin receptor) and α6 (laminin receptor), disrupted VLS formation consistently and revealed that these pathways are necessary conditions for the induction of EM by both NSC lines. In contrast, PDGFRβ (PDGF-BB and -AB receptor) was only required by STROC05 cells, whereas CTXOE03-induced VLS were unaffected by blocking this pathway. As PDGF-BB and -AB secretion in CTXOE03/D3 cocultures was mostly absent, this points to an essential signaling difference between both cell lines. It further indicates that PDGF signaling might not be a necessary condition for VLS formation, but might be sufficient in that it allowed STROC05 cells to be more efficient in VLS formation by stabilizing structures. In STROC05, blocking of PDGF-BB might hence lead to the collapse of VLS, whereas VLS formed by CTXOE03 are not dependent on this support. PDGF-BB secreted from ECs typically recruits pericytes to participate in the formation, stabilization and maturation of vessels^33^ and further supports the notion that STROC05 cells are more efficient “vessel builders”, whereas CTXO03 are more potent vessel destabilizers. Indeed, VLS formed by CTXOE03 were also more easily disrupted by Cilengitide, a αvβ3 blocker, a key element in sprouting angiogenesis^34^. These differences between both NSC lines suggest that CTXOE03 induce a sprouting angiogenesiss, but on their own lack signaling to form stable blood vessels, whereas the signaling profile of STROC05 is more consistent with intussusception and the capability to form stable vessels.

The results presented here allow us to propose a theoretical framework that conceptualizes the cascade of interactions between these two NSC lines and ECs that lead to a neovascularization (Fig. 7B). Neovascularization in the brain is typically caused by hypoxia that activates the angiogenic cascade through hypoxia-inducible factor-1α^35^,^36^. We here hypothesize that in the case of NSC transplantation, as well as our coculture condition, a brief hypoxic condition, caused by the extra metabolic demand of added NSCs, is the trigger of the angiogenic cascade that leads to the release of VEGF-A, which *in vivo* will destabilize existing vessels and *in vitro* produces unstable conditions in a layer of differentiated ECs. MMP-2 plays a crucial role in disrupting the cell-to-cell tight junctions that lead to the breakup of the vessel structure^37^. Both NSC lines here participated in this process, with CTXOE03 being the more potent “activator”, as indicated by the more significant VEGF-A response. This vessel disruption leads to the dedifferentiation and recruitment of EC progenitors to proliferate locally through paracrine factors (BDNF & bFGF)^38^. Indeed, the same factors are involved in NSCs proliferation and demonstrate the similarity and interdependence of signaling between these two cell types^17^,^39^. Once a certain threshold of EC progenitors (and paracrine factors) is reached, EM and the creation of neuropil patches will occur. EM requires guidance from NSCs through Ang-1, PDGF-AB and -BB signaling, contributing to the efficiency of this process, but these pathways are not necessary factors, as demonstrated here in the case of CTXOE03. The deposition of vitronectin and fibronectin provides additional structural support to the tubule formation^40^. At this stage, both TGF-β1 and bFGF are required to stabilize vessels, otherwise these structures will regress.

The formation of novel vessel structures is a complicated process that involves a sequence of signaling events, which exert significant effects on both ECs and NSCs^13^. This conceptual framework provides an opportunity to interfere with or enhance particular molecular interactions between NSCs and ECs in order to determine if these beneficially enhance not only EM (and hence potentially neovascularization), but also NSC differentiation. Although this approach will grant important insights into the communication between these two cell types, it only provides a simplified system to answer relevant questions. To more truthfully represent the *in vivo* condition, additions of pericytes and microglia, as well as a 3D structure potentially supporting flow through lumens are required^41^.

## Conclusion

NSC-induced neovascularization^3^,^11^, the co-transplantation of NSCs and ECs^42^, as well as the potential for tissue-engineered constructs^43^ are dependent on a thorough understanding of the signaling interactions between NSCs and ECs. Uncovering these mechanisms *in vivo* will be very challenging considering the number and nature of factors involved. Modeling these interactions *in vitro* provides a mean to investigate NSCs and ECs interactions, as well as conduct high throughput loss- and gain-of-function experiments. Indeed, we here demonstrated that this *in vitro* coculture assay can be used to measure autocrine/paracrine and juxtacrine factors over time, as well as to perform loss-of-function experiments. Mechanistic *in vitro* studies will be essential to dissect which molecules are driving particular physiological processes and how these relate to the behavioral recovery observed *in vivo*. This type of assay potentially not only affords insights into the molecular mechanisms of these cells’ interaction, but also can provide a method for selecting (unaltered or genetically engineered) cell lines to promote recovery. These types of *in vitro* assays will therefore increasingly become part of defining desirable cell characteristics in our attempts to promote recovery in patients with stroke.

## Methods

All procedures were in accordance with institutional, state and federal guidelines and were approved by the University of Pittsburgh’s human stem cell research oversight (hSCRO) committee (ES-11-006-C Mod 3R). The Institutional Review Board (IRB) determined that there was no involvement of human subjects, according to federal regulations [§ 45 CFR 46.102(f)]

### Monoculture of NSCs and ECs

Human NSC lines STROC05 and CTXOE03 (ECACC accession numbers 04110301 and 04091601, ReNeuron), isolated from the whole ganglionic eminence and the cortex of a 12 weeks’ gestation human fetal brain were cultured^44^,^45^ on laminin-coated plates in serum free medium (Table S4). Both cell lines were produced by transduction with the retroviral vector pLNCX-2 (Clontech) encoding the c-mycER^TAM^ gene. Differentiation was induced by withdrawal of bFGF, EGF, and 4-hydroxytamoxifen (4-OHT).

Human cerebral microvascular endothelial cells (D3, kindly provided by Dr. Pierre-Olivier Couraud, Institut Cochin) were isolated from microvessel fragments of an adult temporal lobe by coexpression of human telomerase reverse transcriptase and the SV40 large T antigen via a lentiviral vector transduction system^46^. D3 were cultured on rat tail collagen type 1 (150 μg/mL, BD Biosciences) in EBM-2 basal medium supplemented with 5% fetal bovine serum (Table S4).

### Coculturing of NSCs and ECs

NSCs were cocultured with ECs using a previously validated model of the neurovascular environment^16^ in order to investigate paracrine and juxtacrine signaling mechanisms underlying EM induced by NSCs. In brief, D3 cells were seeded at a density of 40,000 cells/1.9 cm^2^ in each well of 24-well plates and maintained for 7 days to achieve a density of 400,000 cells/well. Then, NSCs were added at 1,000–1,000,000 cells. Coculture media (Table S4) was replaced daily. For culturing with NSCs that were seeded at a constant density of 40,000 cells, ECs were seeded at densities ranging from 2,000–160,000 cells/well. After 7 days, NSCs were seeded at a density of 40,000 cells/well. After co-culturing for 7 days, cells were fixed with 4% paraformaldehyde for 10 min, rinsed with phosphate buffered saline (PBS) and stored at 4 °C before being processed for immunocytochemistry.

### Immunocytochemistry

Cells were blocked with 10% normal goat serum in PBS containing 0.1% Triton X-100 (Sigma) for 30 min. Cells were incubated with primary antibodies (Table S5) for 18 hrs at 4 °C followed by PBS (3x) washes and appropriate secondary antibodies (Alexa488, Alexa555, Alexa660, 1:1000, Molecular Probes) for 1 hr at 22 °C. Coverslips were rinsed with PBS and mounted in Vectashield with DAPI (Vector). 5 images were taken from 5 different areas at fixed distances from each coverslip using a fluorescent microscope (AxioImager M2, Zeiss). Images were taken in the neuropil or VLS structures (Figure S6A). Within the neuropil a monolayer formed that allowed images to be taken within a single focal plane (Figure S6B) for quantification of colocalization of phenotype markers with DAPI nuclei. For VLS, a 2.5D environment was present, but phenotype markers and DAPI were also readily identified and colocalized within a single focal plane (Figure S6C), but images were taken at the base of the structure, as well as just below the top of the VLS. Quantification was performed on 20x images.

### Vessel-like structure (VLS) formation

To determine the efficiency of inducing endothelial morphogenesis, the total length of segments between branching points of VLS was measured using Stereo Investigator (MBF Bioscience), as previously described^16^.

### Quantification of receptor expression

To determine the level of receptor expression, the light intensity of cells positive for the antibodies was measured using the same settings and exposure time for each coverslip. The light intensity of the cells was calculated as luminosity units (LU) per cell. Cell counts and intensity measurements were performed using ImageJ software (http://rsb.info.nih.gov/ij).

### Enzyme-linked Immuno Sorbent Assay (ELISA)

Cell culture supernatant (500 μL) was collected every 24 hrs and centrifuged at 4,000 rpm for 5 min to remove particulates before being stored at −80 °C. Plain coculture medium served as a control. Soluble factors (Ang-1; Ang-2; BDNF; bFGF; PDGF-BB; PDGF-AB; SDF-1α; TGF-β1; VEGF-A; VEGF-C; VEGF-D; Table S6) were quantified using Quantikine ELISA kits (R&D Systems) on a PowerWave340 microplate spectrophotometer (BioTek).

### Pharmacological blocking of paracrine and juxtacrine signaling

Pharmacological blocking of paracrine factors was introduced from the onset of coculturing using VEGFR2 inhibitor SU1498 (LKT Laboratories), PDGFR inhibitor SU6668 (Santa Cruz), or CXCR4 antagonist AMD3100 (Sigma), at concentrations of 0, 0.5, 1, 5, 10, 25 μM. Juxtacrine factors were blocked using Cilengitide (Medkoo), an inhibitor of integrin αvβ3, at 0, 10, 20, 40, 80 μg/mL. To block integrin α6 signaling, the GoH3 antibody was applied (Beckman Coulter). An IgG_2_A isotype antibody (R&D Systems) served as a control condition. Culture media with inhibitors was changed every other day.

### Statistical Analysis

Immunocytochemistry experiments consisted of 3 biological replicates, each containing 3 technical replicates. ELISAs consisted of 4 biological replicates, each with 2 technical replicates. Most of the data presented here exhibit ceiling or flooring effects, non-parametric statistics were therefore used throughout. A Mann-Whitney U test was performed for two-group comparisons, whereas a Kruskal-Wallis test with Dunn’s post-hoc test was used for multi-groups comparison in Prism 5 (GraphPad).

## Additional Information

**How to cite this article**: Chou, C.-H. and Modo, M. Human neural stem cell-induced endothelial morphogenesis requires autocrine/paracrine and juxtacrine signaling. *Sci. Rep*. **6**, 29029; doi: 10.1038/srep29029 (2016).

## Supplementary Material

Supplementary Information
