# Supplementary material for: Human neural stem cell-induced endothelial morphogenesis requires autocrine/paracrine and juxtacrine signaling
Source: Sci Rep. 2016 Jul 4;6:29029. doi: 10.1038/srep29029 (PMC4931512; doi:10.1038/srep29029)
Supplement: Supplementary Information [file srep29029-s1.pdf]

Supplementary Material:

Human neural stem cell-induced endothelial morphogenesis requires autocrine/paracrine and juxtacrine signaling.

Chung-Hsing Chou[1,2,3] & Michel Modo[1]

[1]University of Pittsburgh, Department of Radiology, Department of Bioengineering, McGowan Institute for Regenerative Medicine, Pittsburgh, USA
[2]Kings College London, Department of Neuroscience, London, UK
[3]Tri-service General Hospital, Department of Neurology, National Defense Medical Centre, Taipei, Taiwan

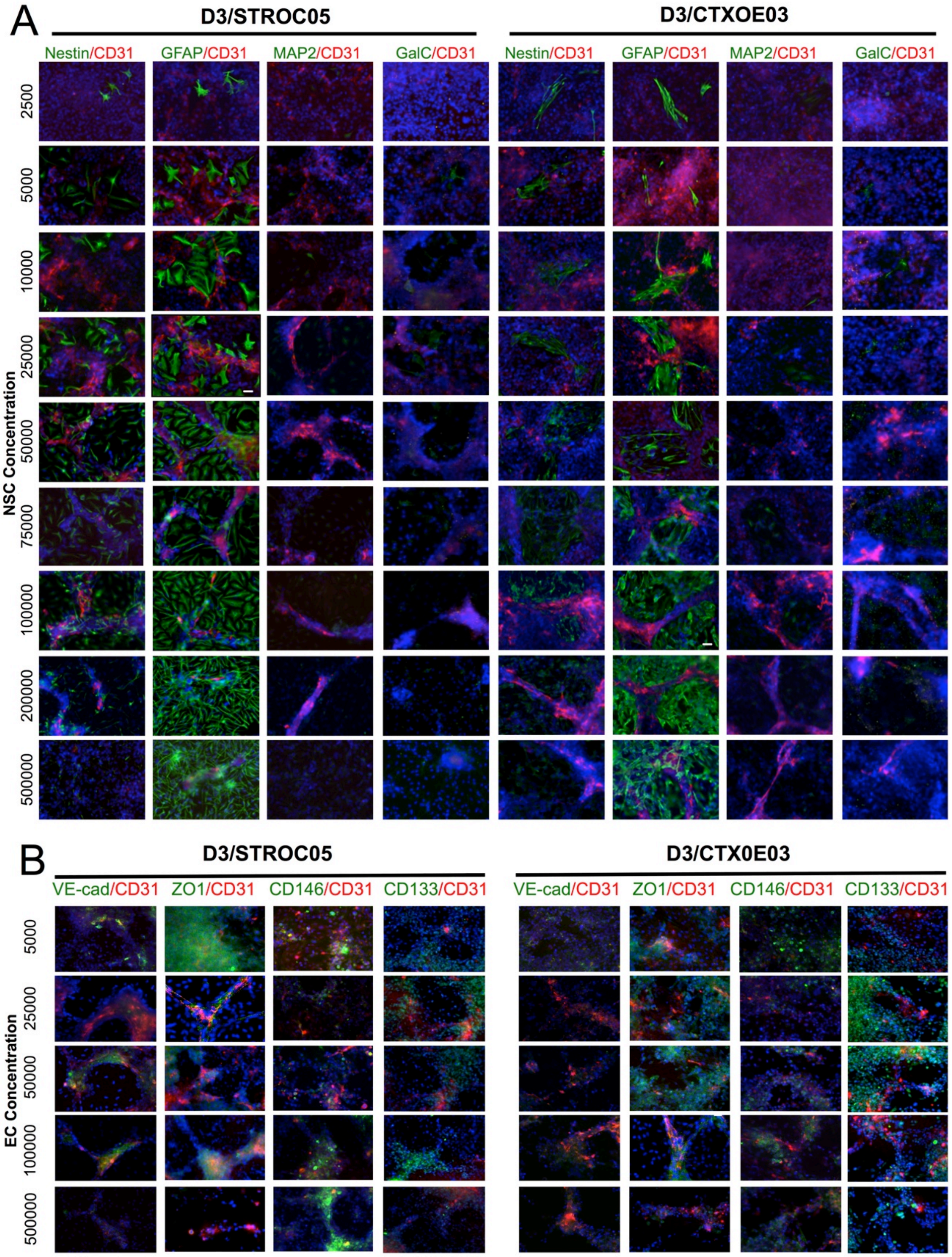

Figure S1. **Immunocytochemical analysis of neural and endothelial markers in NSC/EC cocultures. (A)** Phenotypic characterization of neural markers. **(B)** Phenotypic characterization of endothelial markers. Scale bars represent 50 µm.

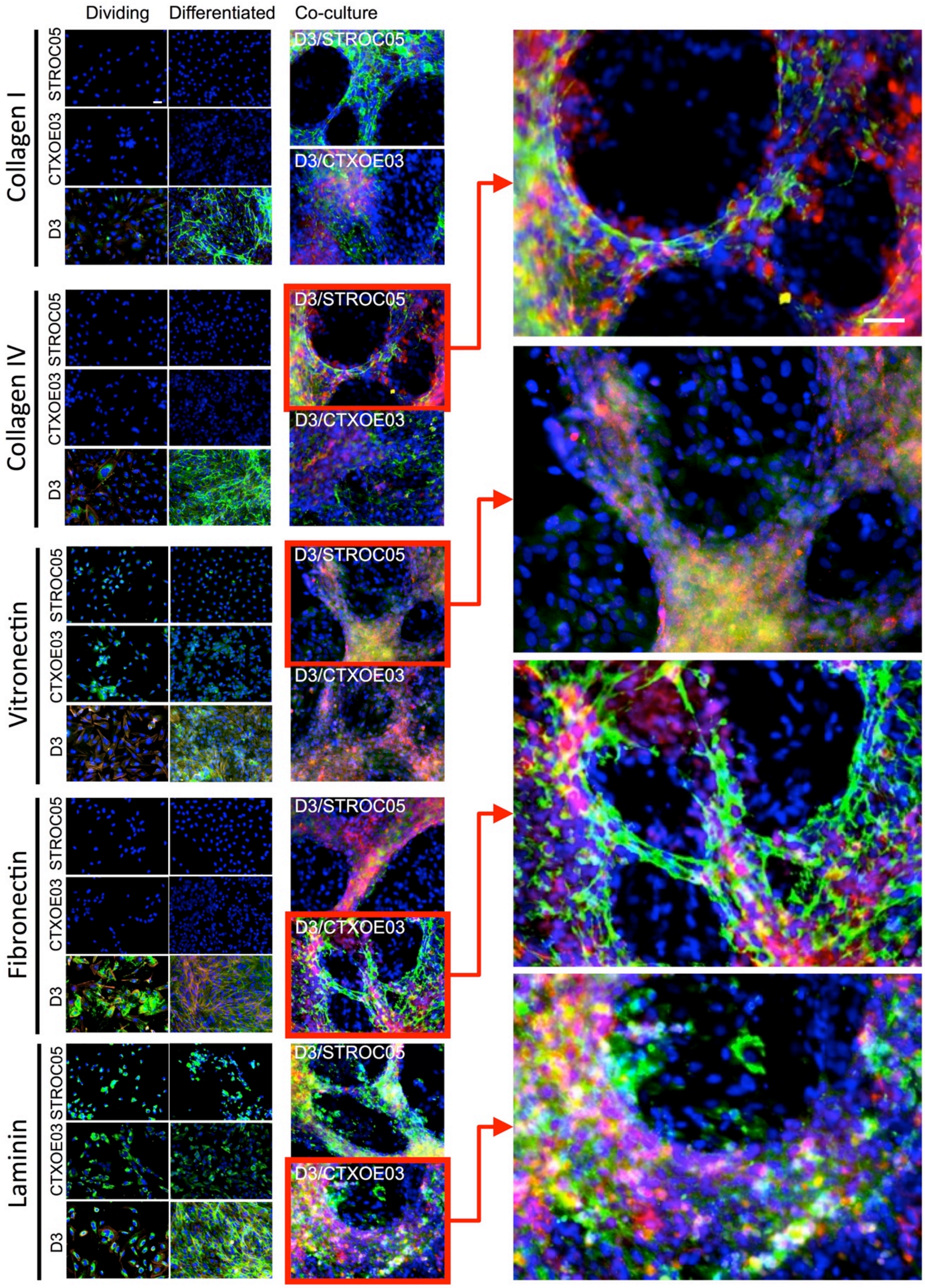

Figure S2. **Immunocytochemical analysis of "vasculature-associated" ECM molecules.** To determine the presence and influence of juxtacrine factors, which are predominantly extracellular matrix associated molecules, STROC05, CTXOE03 and D3, immunohistochemical staining was performed for molecules primarily associated with the vasculature, notably collagen I, collagen IV, vitronectin, fibronectin, laminin. Blue=DAPI; Green=ECM molecule; Red=CD31; Phalloidin (orange). Scale bars represent 50 μm.

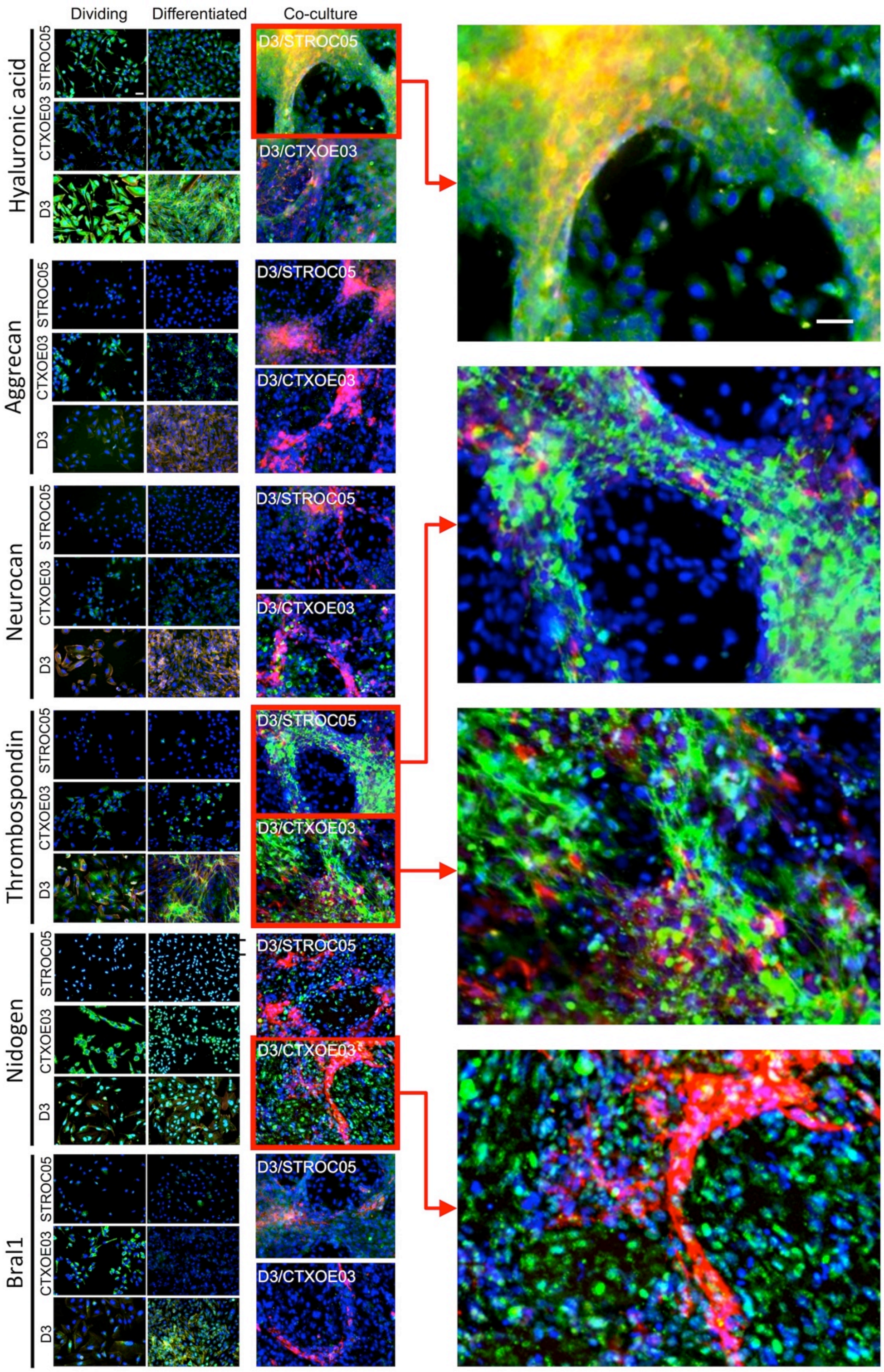

Figure S3. **Immunocytochemical analysis of "neuropil-associated" ECM molecules.** To determine the presence and influence of juxtacrine factors which are predominantly extracellular matrix associated molecules, STROC05, CTXOE03 and D3, immunohistochemical staining was performed for molecules primarily associated with the neuropil, notably hyaluronic acid, aggrecan, neurocan, thrombospondin, nidogen and brain-link protein 1 (Bral1). Phalloidin (orange) used as a counterstain for D3 monoculture. Blue=DAPI; Green=ECM molecule; Red=CD31. Scale bars represent 50 µm.

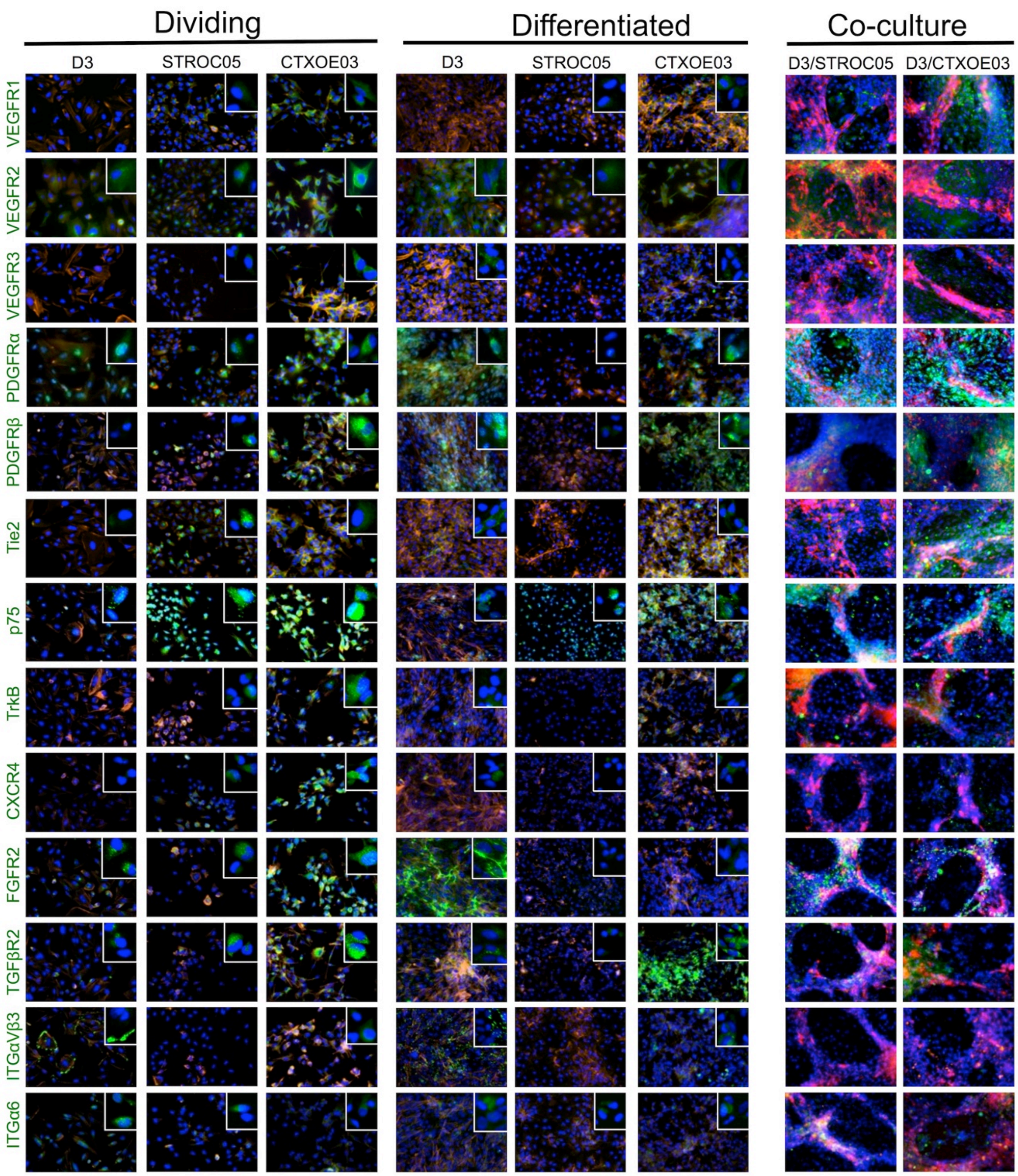

Figure S4. **Immunocytochemistry of receptor expression**. Blue=DAPI; Green=Receptor; Red=CD31. Phalloidin (orange) binds F-actin, serving as a counterstain for monoculture of all three cell lines. Scale bars represent 50 μm.

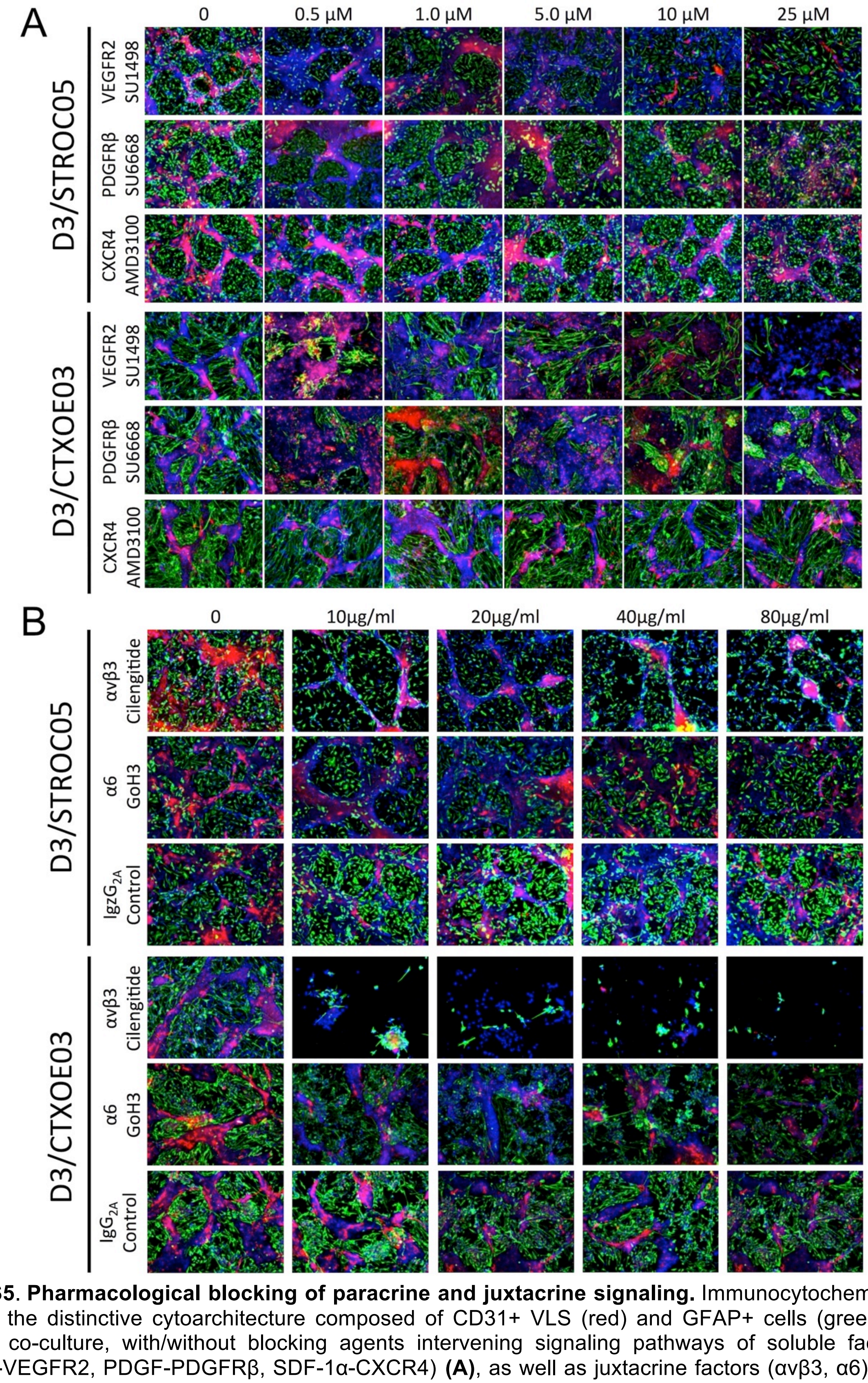

Figure S5. **Pharmacological blocking of paracrine and juxtacrine signaling.** Immunocytochemistry revealed the distinctive cytoarchitecture composed of CD31+ VLS (red) and GFAP+ cells (green) in NSC/EC co-culture, with/without blocking agents intervening signaling pathways of soluble factors (VEGFA-VEGFR2, PDGF-PDGFRβ, SDF-1α-CXCR4) **(A)**, as well as juxtacrine factors (αvβ3, α6) **(B)**. IgG2A isotype antibody served as a control for GoH3-blocking antibody against integrin α6. Scale bars represent 200 μm.

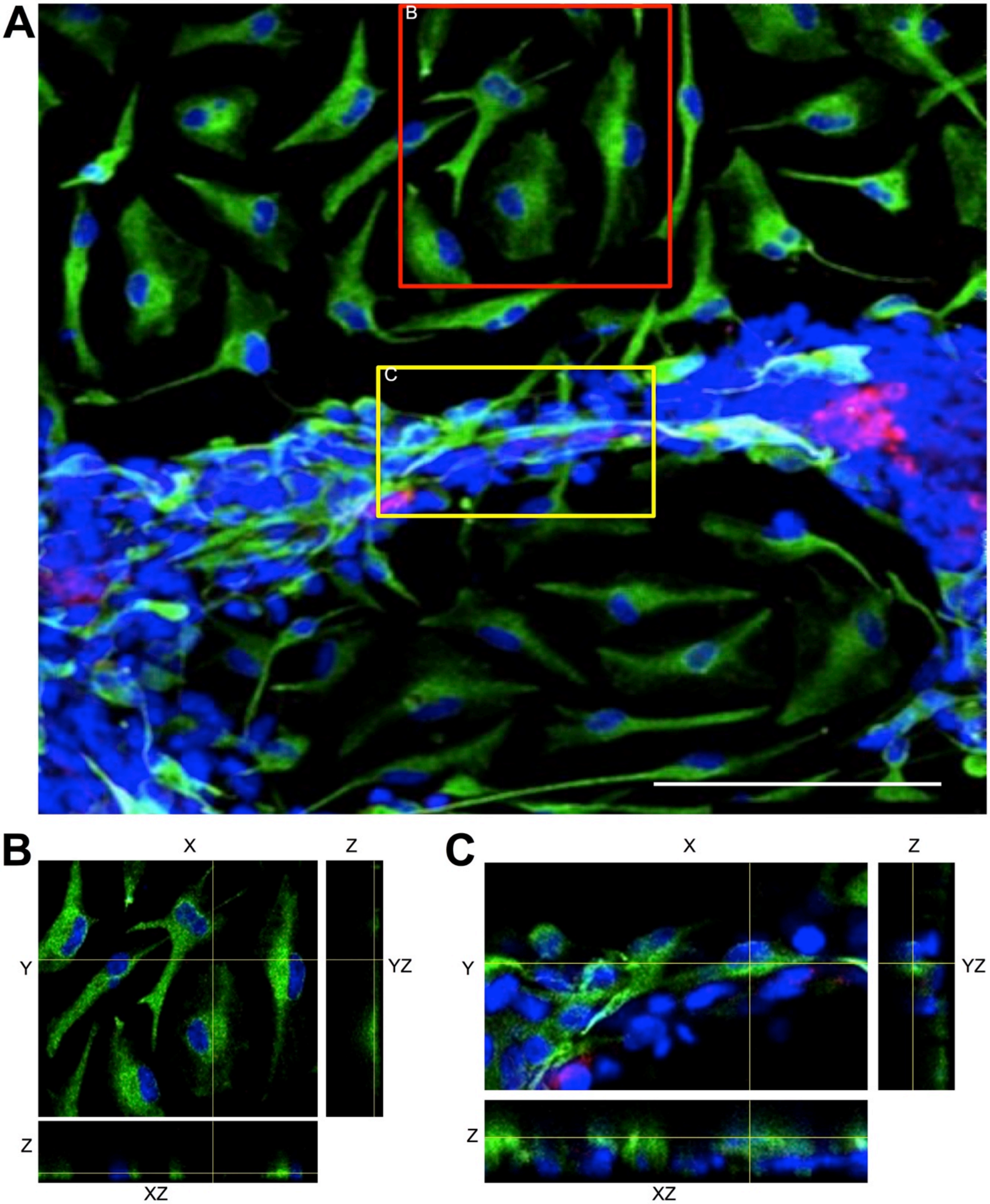

Figure S6. **Co-localization analysis of immunocytochemical markers. (A)** A maximum projection image of a 3D confocal image stack consisting of 36 individual images in the z direction demonstrates a “neuropil” environment that is a monolayer of unmistakably separated and defined cells consisting exclusively of NSCs. **(B)** Phenotypic characterization within this monolayer was very straightforward using a co-localization of the phenotypic markers with a DAPI-labeled nucleus for cell quantification. **(C)** Within and around the VLS, a 2.5D environment was created by both NSCs and ECs. Co-localization of phenotypic markers to individual cell nuclei within these VLS was also feasible using images from a single focal plane. z stacks for cell counting were hence not required. An inter- and intra-rater reliability of > 95% was achieved. Scale bar represents 100 μm.

SUPPLEMENTARY TABLES

Marker	Cell Line	Seeding Density	Cell Line x Seeding Density
Nestin	F(1;19)=3.93, n.s.	F(4;19)=8.97***	F(4;19)=2.92*
MAP2	F(1;20)=34.42****	F(4;20)=4.45**	F(4;20)=7.53***
GalC	F(1;20)=11.9**	F(4;20)=51.98****	F(4;20)=25.49****
GFAP	F(1;20)=1.95, n.s.	F(4;20)=9.14***	F(4;20)=1.78, n.s.
CD31	F(1;20)=0.09, n.s.	F(4;20)=16.12****	F(4;20)=11.37****
VEcad	F(1;20)=46.79****	F(4;20)=8.26***	F(4;20)=1.42, n.s.
ZO1	F(1;20)=21.15***	F(4;20)=70.12****	F(4;20)=46.6****
CD146	F(1;20)=7.4*	F(4;20)=3.14*	F(4;20)=5.37**
CD133	F(1;20)=3.97, n.s.	F(4;20)=1.45, n.s.	F(4;20)=5.13**

Table S1. Associations between cell line, seeding density and the expression of antigens. Two-way ANOVA tests for interpreting influence of the cell line and the seeding density on expression of differentiation markers in EC/NSC co-cultures. Results of Bonferroni post-hoc tests are shown in Figure 3. (*) p<0.05, (**) p<0.01, (***) p<0.001, (****) p<0.0001.

Soluble Factor	Cell Line	Time	Cell Line x Time
BDNF	F(1;10)=9.3*	F(4;10)=3.21, n.s.	F(4;10)=2.91, n.s.
SDF-1α	F(1;10)=31.97***	F(4;10)=0.98, n.s.	F(4;10)=2.6, n.s.
VEGFA	F(1;10)=1451.44****	F(4;10)=391.56****	F(4;10)=274.6****
VEGFC	F(1;10)=384.71****	F(4;10)=12.39***	F(4;10)=6.09**
PDGF-BB	F(1;10)=519.92****	F(4;10)=0.69, n.s.	F(4;10)=1.61, n.s.
PDGF-AB	F(1;10)=719.02****	F(4;10)=62.71****	F(4;10)=3.15, n.s.
ANG-1	F(1;10)=632.31****	F(4;10)=127.18****	F(4;10)=201.33, n.s.
ANG-2	F(1;10)=181.73****	F(4;10)=9.31**	F(4;10)=8.78**
TGFb1	F(1;10)=1.32, n.s.	F(4;10)=15.19***	F(4;10)=3.17, n.s.
bFGF	F(1;10)=290.65****	F(4;10)=5.46*	F(4;10)=2.01, n.s.

Table S2. Associations between cell line, culture duration and concentrations of soluble factors. Two-way ANOVA tests were used for interpreting the significance of the cell line and the culture duration on concentrations of soluble factors in EC/NSC co-cultures. (brain-derived neurotrophic factor = BDNF, basic fibroblast growth factors = bFGF, platelet-derived growth factor = PDGF, stromal cell-derived factor = SDF, transforming growth factor = TGF, vascular endothelial growth factor = VEGF) (*) p<0.05, (**) p<0.01, (***) p<0.001, (****) p<0.0001.

EC/NSC co-culture	Drug	Concentration	Drug x Concentration
Blockade of soluble factors			
D3/STR	F(2;36)=14.11****	F(5;36)=26.2****	F(10;36)=4.94***
D3/CTX	F(2;36)=23.33****	F(5;36)=18.32****	F(10;36)=12.56****
Blockade of contact-mediated factors			
D3/STR	F(2;55)=45.54****	F(4;55)=20.78****	F(8;55)=4.06***
D3/CTX	F(2;48)=222.1****	F(4;48)=50****	F(8;48)=16.12****

Table S3. Associations between anti-angiogenic agents, agent concentrations and total length of capillary-like structures. Two-way ANOVA tests were used for interpreting the significance of the types and concentrations of anti-angiogenic agents on formation of capillary-like structures in EC/NSC co-cultures. (*) p<0.05, (**) p<0.01, (***) p<0.001, (****) p<0.0001.

Component	D3 medium	STROC05 medium	CTXOE03 medium	D3/STROC05 co-culture medium	D3/CTXOE03 co-culture medium	Supplier	Cat. Ref.
DMEM:F12		Basal medium		Basal medium (half volume)		Sigma-Aldrich	D6421
EBM-2	Basal medium			Basal medium (half volume)		Lonza	CC-3156
Fetal bovine serum	5%					PAA	A15-151
Hydrocortisone	1.4 µM			0.7 µM		Sigma-Aldrich	H-0135
Acid ascorbic	5 µg/mL			2.5 µg/mL		Sigma-Aldrich	A4544
Chemically defined lipid concentrate	1%			0.5%		Invitrogen	11905-031
HEPES	10 mM			5 mM		Sigma-Aldrich	83264
Human albumin solution		0.03%		0.015%		GemBio	800-121
Transferrin, human		100 µg/mL		50 µg/mL		Sigma-Aldrich	T1147
Putrescine DiHCl		16.2 µg/mL		8.1 µg/mL		Sigma-Aldrich	P5780
Insulin, human		5 µg/mL		2.5 µg/mL		Sigma-Aldrich	I9278
Progesterone		60 ng/mL		30 ng/mL		Sigma-Aldrich	P8783
L-glutamine		2 mM		1 mM		Sigma-Aldrich	G7513
Sodium selenite		40 ng/mL		20 ng/mL		Sigma-Aldrich	S9133
L-thyroxine (T4)		400 ng/mL		200 ng/mL		Sigma-Aldrich	T0397
Tri-iodo-thyronine (T3)		337 ng/mL		118.5 ng/mL		Sigma-Aldrich	T6397
Heparin sodium		10 Units/mL		5 Units/mL		Sigma-Aldrich	H3149
Corticosterone		40 ng/mL		20 ng/mL		Sigma-Aldrich	C2505
*bFGF	1 ng/mL	10 ng/mL				PeproTech	AF-100-18B
*EGF		20 ng/mL				PeproTech	AF-100-15
*4-hydroxytamoxifen		100 nM				Sigma-Aldrich	H7904

Table S4. Composition of culture media. Composition of cell culture media for D3 human cerebral microvascular endothelial cell line, STROC05 and CTXOE03 human neural stem cell lines, and EC/NSC co-culture media. Factors with a * were removed to induce cell differentiation.

Antigen	Antibody	Conc.	Company	Cat. Ref.
Aggrecan	Mouse anti-aggrecan	1:250	Abcam	ab3778
BRAL1	Rabbit anti-brain-specific link protein 1	1:400	Sigma	PRS4501
CD31/ PECAM-1	Mouse anti-platelet endothelial cell adhesion molecule 1	1:100	Santa Cruz	sc-13537
CD31/ PECAM-1	Rabbit anti-platelet endothelial cell adhesion molecule 1	1:200	Abcam	ab28364
CD133/ Prominin-1	Mouse anti-CD133	1:300	Millipore	MAB4399
CD144/ VE-cadherin	Mouse anti-vascular endothelial-cadherin	1:200	Abcam	ab7047
CD146/ MCAM	Mouse anti-melanoma cell adhesion molecule	1:300	Abcam	ab24577
Collagen I	Rabbit anti-collagen type I	1:500	Abcam	ab292
Collagen IV	Goat anti-collagen type IV	1:200	Millipore	ab769
CXCR4	Mouse anti-CXC-chemokine receptor 4	1:200	Abcam	ab45001
FGFR2	Mouse anti FGF receptor 2	1:400	Abcam	ab58201
Fibronectin	Mouse anti-fibronectin	1:200	Abcam	ab6328
GalC	Mouse anti-galactocerebroside	1:200	Millipore	MAB342
GFAP	Mouse anti-glial fibrillary acid protein	1:3000	Sigma	G3893
Hyaluronic acid	Sheep anti-hyaluronic acid	1:100	Abcam	ab53842
ITG-α6	Mouse anti-integrin alpha 6A	1:200	Millipore	MAB1356
ITG-αvβ3	Mouse anti-integrin alpha v beta 3	1:250	Abcam	ab78289
Ki67	Rabbit anti-Ki67	1:500	Abcam	ab15580
Laminin	Chicken anti-laminin	1:500	Abcam	ab14055
MAP2	Mouse anti-microtubule associate protein-2	1:500	Abcam	ab11267
NID1	Mouse anti-nidogen	1:250	Sigma	sab1400185
Nestin	Mouse anti-nestin	1:500	Millipore	MAB5326
Nestin	Rabbit anti-nestin	1:1000	Abcam	ab5968
Neurocan	Mouse anti-neurocan	1:250	Abcam	ab31979
p75	Rabbit anti-NGF receptor p75	1:400	Millipore	AB1554
PDGFRα	Rabbit anti-PDGF receptor alpha	1:100	Abcam	ab61219
PDGFRβ	Rabbit anti-PDGF receptor beta	1:200	Abcam	ab32570
SOX2	Goat anti-sex determining region Y-box 2	1:200	Santa Cruz	sc-17320
TGFβR2	Mouse anti TGF beta receptor II	1:200	Abcam	ab78419
Thrombospondin	Mouse anti-thrombospondin	1:200	Abcam	ab1823
Tie2	Mouse anti-tunica intima endothelial kinase 2	1:100	Abcam	ab24859
TrkB	Rabbit anti-TrkB	1:200	Abcam	ab131483
VEGFR1	Mouse anti-VEGF receptor 1	1:50	Abcam	ab9540
VEGFR2	Mouse anti-VEGF receptor 2	1:100	Abcam	ab9530
VEGFR3	Mouse anti-VEGF receptor 3	1:100	Abcam	ab51496
Vitronectin/ ligand for ITG-αvβ3	Mouse anti-vitronectin	1:1000	Abcam	ab13413
ZO1	Rabbit anti-zonula occludens 1	1:500	Zymed	40-2200

Table S5. Primary antibodies. Evaluation of undifferentiated and differentiated cells as well as capillary-like structure was achieved by immunocytochemistry against antigens that were present on D3, STROC05, and CTXOE03 cells. (brain-specific link protein 1 = BRAL1, platelet endothelial cell adhesion molecule 1 = PECAM-1, vascular endothelial-cadherin = VE-cadherin, CXC-chemokine receptor 4 = CXCR4, fibroblast growth factor = FGF, galactocerebroside = GalC, glial fibrillary acid protein = GFAP, integrin = ITG, melanoma cell adhesion molecule = MCAM, microtubule associate protein-2 = MAP2, nerve growth factor = NGF, nidogen = NID1, platelet-derived growth factor = PDGF, sex determining region Y-box 2 = SOX2, transforming growth factor = TGF, tunica intima endothelial kinase 2 = Tie2, vascular endothelial growth factor = VEGF, zonula occludens 1 = ZO1).

Soluble factor	MDD (pg/mL)	Cat. Ref. (R&D Systems)
Angiopoietin-1	3.45	DANG10
Angiopoietin-2	8.2	DANG20
BDNF	<20	DBD00
bFGF	<3	DFB50
PDGF-AB	1.14	DHD00C
PDGF-BB	<15	DBB00
SDF-1α	18	DSA00
TGFβ1	4.61	DB100B
VEGFA	<5	DVE00
VEGFC	13.3	DVEC00
VEGFD	11.4	DVED00

Table S6. Quantikine ELISA kits. Measurement and quantification of soluble factors in supernatants of monoculture and co-culture of ECs and NSCs was conducted, using solid phase colorimetric sandwich Quantikine ELISA kit for human (R&D Systems). (minimum detectable dose = MDD, brain-derived neurotrophic factor = BDNF, basic fibroblast growth factors = bFGF, platelet-derived growth factor = PDGF, stromal cell-derived factor = SDF, transforming growth factor = TGF, vascular endothelial growth factor = VEGF).
